# A Nationwide Census of ICU Capacity and Admissions in Mongolia

**DOI:** 10.1371/journal.pone.0160921

**Published:** 2016-08-17

**Authors:** Naranpurev Mendsaikhan, Tsolmon Begzjav, Ganbold Lundeg, Andreas Brunauer, Martin W. Dünser

**Affiliations:** 1 Intensive Care Department, Intermed Hospital, Ulaanbaatar, Mongolia; 2 Division of Emergency Medicine and Anesthesia, Health Sciences University of Mongolia, Ulaanbaatar, Mongolia; 3 Department of Anesthesiology, Perioperative Medicine and General Intensive Care Medicine, Salzburg University Hospital and Paracelsus Private Medical University, Salzburg, Austria; Columbia University, UNITED STATES

## Abstract

In Mongolia, a Central Asian lower-middle income country, intensive care medicine is an under-resourced and–developed medical specialty. The burden of critical illness and capacity of intensive care unit (ICU) services in the country is unknown. In this nationwide census, we collected data on adult and pediatric/neonatal ICU capacities and the number of ICU admissions in 2014. All hospitals registered to run an ICU service in Mongolia were surveyed. Data on the availability of an adult and/or pediatric/neonatal ICU service, the number of available ICU beds, the number of available functional mechanical ventilators, the number of patients admitted to the ICU, and the number of patients admitted to the study hospital were collected. In total, 70 ICUs with 349 ICU beds were counted in Mongolia (11.7 ICU beds/100,000 inhabitants; 1.7 ICU beds/100 hospital beds). Of these, 241 (69%) were adult and 108 (31%) pediatric/neonatal ICU beds. Functional mechanical ventilators were available for approximately half of the ICU beds (5.1 mechanical ventilators/100,000 inhabitants). While all provincial hospitals ran a pediatric/neonatal ICU, only dedicated pediatric hospitals in Ulaanbaatar did so. The number of adult and pediatric/neonatal ICU admissions varied between provinces. The number of adult ICU beds and adult ICU admissions per 100,000 inhabitants correlated (*r* = 0.5; *p* = 0.02), while the number of pediatric/neonatal ICU beds and pediatric/neonatal ICU admissions per 100,000 inhabitants did not (*r* = 0.25; *p* = 0.26). In conclusion, with 11.7 ICU beds per 100,000 inhabitants the ICU capacity in Mongolia is higher than in other low- and lower-middle-income countries. Substantial heterogeneities in the standardized ICU capacity and ICU admissions exist between Mongolian provinces. Functional mechanical ventilators are available for only half of the ICU beds. Pediatric/neonatal ICU beds make up one third of the national ICU capacity and appear to meet or even exceed the demand of pediatric/neonatal critical care.

## Introduction

Despite of a high burden of critical illness in lower-middle-income and low-income countries [1,2], management of critically ill patients faces serious challenges in these regions of the world. The few intensive care unit (ICU) services that exist are often hampered by a shortage of bed capacities, physicians and nurses with specific training in intensive care, as well as material resources [3,4]. This often results in inadequate care associated with high mortality rates and adverse functional long-term outcomes [5–7]. Intensive care medicine in Mongolia is a young and under-developed medical specialty [8,9]. While studies have shown similar limitations in adequately trained ICU staff and material resources as reported in other resource restricted settings [8,9], no data on the capacity of ICU beds and the burden of critical illness in Mongolia exist so far. Knowledge of the availability of ICU beds and their distribution in the country could help to identify and prioritize strategies to improve the care of critically ill patients in Mongolia.

In this nationwide census we sought to determine the ICU capacity and number of ICU admissions for adults and children in Mongolia in 2014.

## Materials and Methods

A nationwide census of all hospitals with an ICU in Ulaanbaatar (capital city with provincial status) and the 21 provinces of Mongolia was performed in October/November 2015. The study protocol was approved by the Ethics Committee of the Medical University of Mongolia. As only anonymized data were collected, no changes to patient care were made, and hospital participation was voluntary, no written informed patient consent was required.

### Intensive Care Medicine in Mongolia

Mongolia is a land-locked Central Asian country which is home to approximately three million people and classified as a lower-middle-income country. The Mongolian population has a life expectancy of 69.3 years at birth (median age 27.5 years) and annually grows by 1.3% (20.3 births/1,000 inhabitants). Seventy-two percent of Mongolians live in cities; 35 and 40% of the total population have inadequate drinking water and sanitation facility access. In spite of an economic boom after the end of the communist rule in 1990, Mongolia continues to encounter substantial health care problems. Six percent of the gross domestic product is spent on health care. The physician and hospital bed density per 1,000 inhabitants was 2.8 and 6.8 in 2011 and 2012, respectively [10]. The top five causes of death include ischemic heart disease (22.4% of all deaths), stroke (16.9%), liver cancer (7.6%), liver disease (5.1%) and lower respiratory tract infections (4.1%) [11].

Primary level hospitals provide general medical care for the Mongolian population at a county level. All critically patients admitted to a primary level hospital are transferred to a secondary level hospital which is the only hospital in the province with an ICU service. Secondary level hospitals, both in the provinces and Ulaanbaatar, offer comprehensive but no specialist health services. Specialist health services in Mongolia are provided exclusively by tertiary level or private hospitals which are all located in the capital city. In Ulaanbaatar, which is home to approximately 45% of the Mongolian population and has the highest density of hospitals in the country, all level three referral hospitals and a selected number of private and secondary level hospitals run ICU services. The private hospitals have <100 beds, are administered by overseas companies and offer selected specialist services. Most of the ICUs in Mongolia are run by anaesthetists or pediatricians. Specialized ICU physician teams exist in only few hospitals. Given a state-wide insurance system, ICU admission is not limited by financial barriers.

### Data Collection

All hospitals registered at the Mongolian Ministry of Health to run an ICU service were visited or the medical director of the ICU was contacted by phone. The following data were collected during the time period between January 1 and December 31, 2014: availability of an adult and/or pediatric/neonatal ICU service, the number of available ICU beds, the number of available functional mechanical ventilators, the number of patients admitted to the ICU, and the number of patients admitted to the study hospital. The following data (referring to the same time period) were collected from the national statistical institute for all Mongolia as well as separately for each province and Ulaanbaatar: the number of inhabitants, the number of hospital beds (including also those hospitals without an ICU), and the number of hospital admissions (including also admission to those hospitals without an ICU). Based on these figures the number of ICU beds, mechanical ventilators and ICU admissions per 100,000 inhabitants as well as ICU beds per 100 hospital beds were calculated for all Mongolia and separately for each province as well as for adult and pediatric/neonatal ICU services.

### Statistical Analysis

All statistical analyses were performed using the SPSS 20 statistical software package (IBM, Germany). The anonymized data used for the statistical analysis are provided as a supporting information to this manuscript (S1 File). Descriptive methods were applied to present absolute numbers, percentages and ratios. Comparisons of ICU capacities between the provinces and Ulaanbaatar were performed using the Mann Whitney-*U*, Chi²- or Fisher’s Exact test, as appropriate. The relationship between the number of ICU beds and ICU admissions per 100,000 inhabitants was determined with the use of a bivariate correlation analysis applying the Pearson correlation coefficient. All data are given as median values with interquartile ranges, if not otherwise indicated. *P*-values <0.05 were considered to indicate statistical significance.

## Results

**Fig 1:**
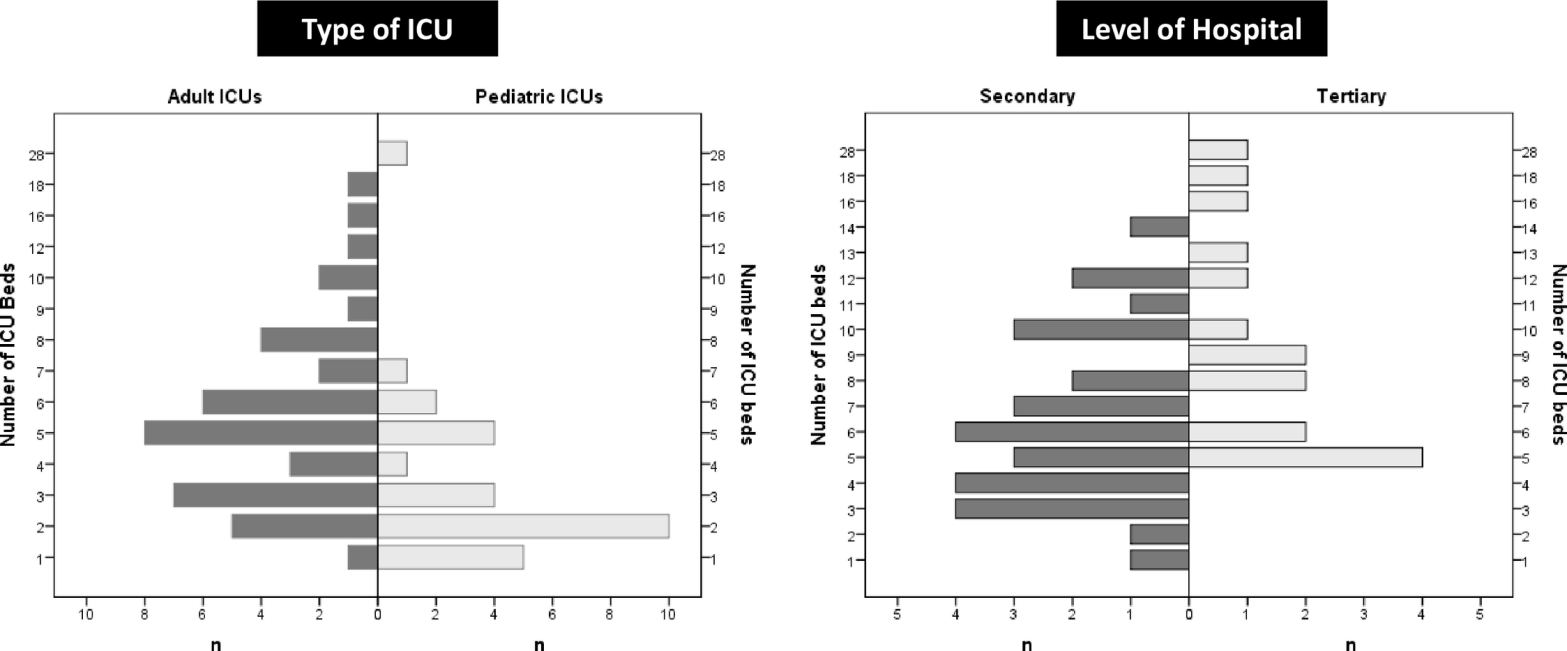
Number of beds per intensive care unit for adult and pediatric services as well as for secondary and tertiary level hospitals. ICU, intensive care unit.

**Fig 2:**
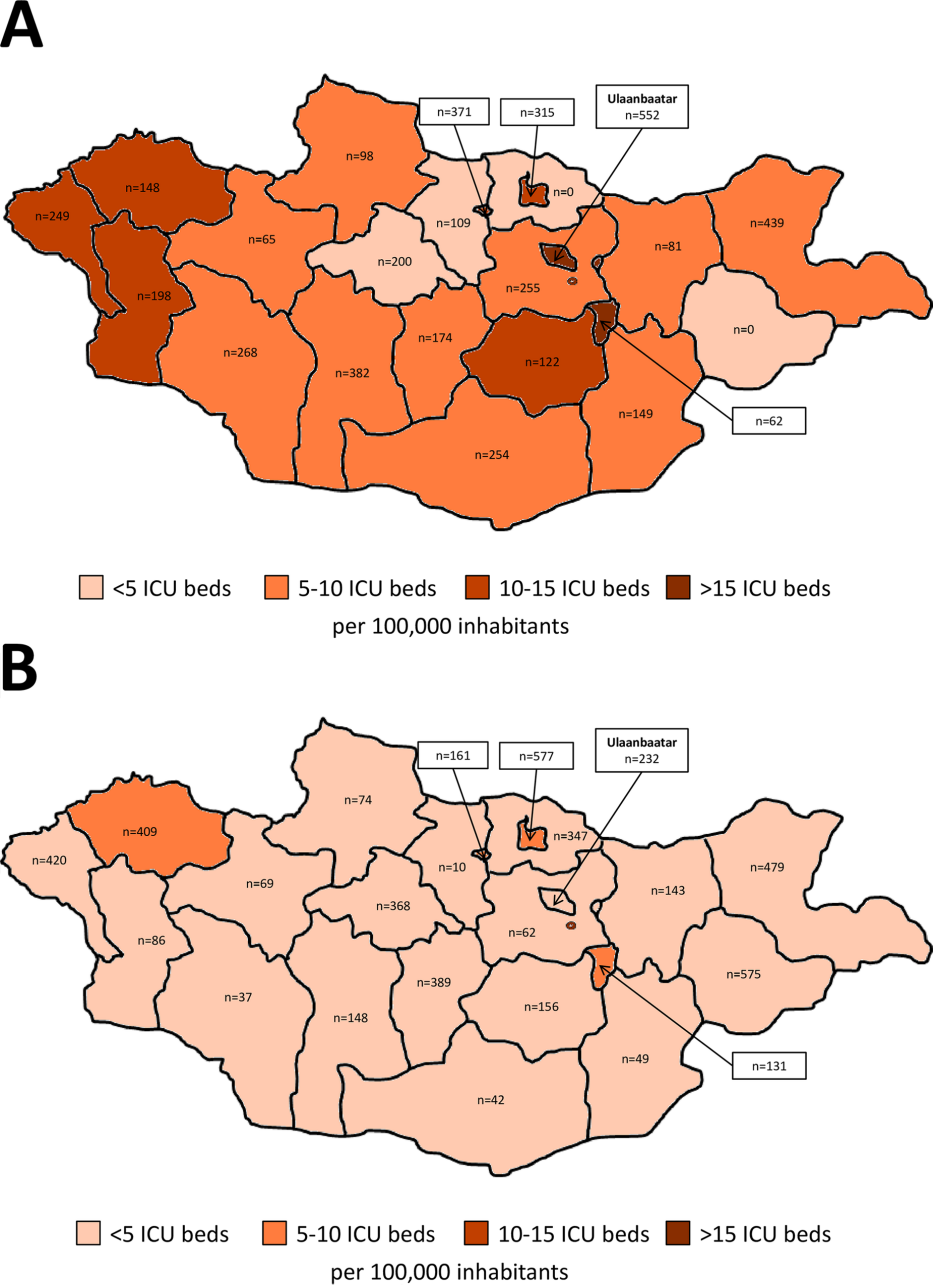
Number of intensive care unit admissions per 100,000 inhabitants and intensive care unit beds per 100,000 inhabitants in Ulaanbaatar and the provinces. (A) adult data. (B) pediatric/neonatal data. ICU, intensive care unit.

In total, seventy ICUs with 349 ICU beds were counted in Mongolia. Of these ICU beds, 241 (69%) were dedicated for adults and 108 (31%) for children. Adult ICUs in Ulaanbaatar had more mechanical ventilators than adult ICUs in the provinces. The number of ICU beds per pediatric/neonatal ICU was higher in Ulaanbaatar than in the provinces. While all provincial hospitals ran a pediatric/neonatal ICU, only dedicated pediatric hospitals in Ulaanbaatar did so (Table 1). There was a wide variation in the number of beds per ICU for adult and pediatric ICUs as well as per ICU in secondary and tertiary hospitals (Table 2, Fig 1). The number of adult and pediatric/neonatal ICU admissions varied between provinces (Table 3, Fig 2). While the number of adult ICU beds and adult ICU admissions per 100,000 inhabitants correlated (Pearson correlation coefficient, 0.5; *p* = 0.02), the number of pediatric/neonatal ICU beds and pediatric/neonatal ICU admissions per 100,000 inhabitants did not (Pearson correlation coefficient, 0.25; *p* = 0.26).

**Table 1:** Availability of intensive care units, intensive care unit beds, and mechanical ventilators.

Parameter	Total	Provinces	Ulaanbaatar	*p*-value
Inhabitants (n)	2,990,617	1,642,975	1,347,642	
Hospitals with an ICU (absolute n)	45	24	26	
Hospital level (n/%)				0.06
*Level II*	29 (64.4)	17 (81)	12 (50)	
*Level III*	16 (35.6)	4 (19)	12 (50)	
Hospital type (n/%)				0.24
*governmental*	42 (93.3)	21 (100)	21 (87.5)	
*private*	3 (6.7)	0 (0)	3 (12.5)	
Median hospital bed no (n)	225 (154–301)	212 (170–276)	238 (128–344)	0.65
Adult ICU (n/%)	42 (93.3)	19 (90.5)	23 (95.8)	0.59
Median adult ICU beds (n)	5 (3–7)	4 (3–6)	6 (4–9)	0.3
Median adult ventilators (n)	2 (1–3)	2 (1–2)	3 (2–4)	0.04*
Pediatric/Neonatal ICU (n/%)	28 (62.2)	21 (100)	7 (29.2)	<0.001*
Median pediatric/neonatal ICU beds (n)	2 (2–5)	2 (1.5–3)	5 (3–5)	0.01*
Median pediatric/neonatal ventilators (n)	1 (0–1)	1 (0–1)	1 (0–2.5)	0.18

ICU, intensive care unit; *p*-values refer to comparisons between the provinces and Ulaanbaatar;

*, significant difference between the provinces and Ulaanbaatar.

**Table 2:** Availability of intensive care unit beds and functional mechanical ventilators per 100.000 inhabitants for each Mongolian province.

Province	Inhabitants	ICU beds/100,000 inhabitants	ICU beds/100 hospital beds	MVs/100,000 inhabitants	Adult ICU beds/100,000 inhabitants	Adult MVs/100,000 inhabitants	Pediatric ICU beds/100,000 inhabitants	Pediatric MVs/100,000 inhabitants
**All Mongolia**	**2,990,617**	**11.7 (1.7–18.7)**	**1.7 (0.3–3.5)**	**5.1 (0.9–18.7)**	**8.1 (0–12.5)**	**3.6 (0–12.5)**	**3.6 (1.6–9.2)**	**1.5 (0–6.2)**
Arhangai	93,086	4.3	0.8	4.3	2.2	1.1	2.2	3.2
Bayanhongor	83,044	9.6	1.7	2.4	7.2	1.2	2.4	1.2
Bayanulgii	95,151	12.6	1.8	5.3	8.4	4.2	4.2	1
Bulgan	60,494	3.3	0.6	5	1.7	3.3	1.7	1.7
Darkhan Uul	99,947	14	2.1	6	8	4	6	2
Dornod	75,194	9.3	1.6	8	6.7	5.3	2.7	2.7
Dornogovi	63,808	6.4	1	3.1	4.7	3.1	1.6	0
Dundgovi	44,351	13.7	2.6	6.8	11.5	2.3	2.3	4.5
Govi altai	56,735	8.8	1.3	5.3	5.3	3.5	3.5	1.8
Govi Sumber	16,058	18.7	3.5	18.7	12.5	12.5	6.2	6.2
Hovd	81,479	11	1.6	3.7	7.4	2.5	3.7	1.2
Huvsgul	126,043	5.6	1	1.6	4	0.8	1.6	0.8
Khentii	71,212	5.6	1	4.2	2.8	1.4	2.8	2.8
Orkhon	94,421	13.8	2.2	4.2	7.4	2.1	6.4	2.1
Selenge	106,212	2.8	0.5	0.9	0	0	2.8	0.9
Sukhbaatar	57,423	1.7	0.3	1.7	0	1.7	1.7	0
Tuv	90,107	6.7	1.2	3.3	4.4	2.2	2.2	1.1
Ulaanbaatar (capital city)	1,347,642	15.7	2	6.2	11.7	5	4	1.2
Umnugovi	69,694	7.2	1.1	5.7	4.3	2.9	2.9	2.9
Uvs	75,792	14.5	2.1	6.6	5.3	4	9.2	2.64
Uvurhangai	112,992	7.1	1.2	2.7	5.3	1.8	1.8	0.9
Zavkhan	69,732	7.2	1.1	1.4	4.3	1.4	2.9	0

ICU, intensive care unit; MV, mechanical ventilator. All data referring to provinces are given as absolute numbers. Data for all Mongolia additionally includes ranges between minimum and maximum values in parentheses.

**Table 3:** Adult and pediatric/neonatal intensive care unit admissions in Mongolia in 2014.

Parameter	All Mongolia	Provinces	Ulaanbaatar
Hospital admissions (hospitals with an ICU) (n)	448,581	182,964	265,617
Hospital admissions (all hospitals) (n)	747,973	355,173	392,800
Adult ICU admissions (n)	10,676	3,237	7,439
Adult ICU admissions (% of admissions in hospitals with an ICU)	2.38	1.77	2.8
Adult ICU admissions (% of all hospital admissions*)	1.43	0.91	1.89
Adult ICU admissions per 100,000 inhabitants (n)	357	197	552
Pediatric/neonatal ICU admissions (n)	7,073	3,949	3,124
Pediatric/neonatal ICU admissions (% of admissions in hospitals with an ICU)	1.58	2.16	1.18
Pediatric/neonatal ICU admissions (% of all hospital admissions*)	0.95	1.11	0.8
Pediatric/neonatal ICU admissions per 100,000 inhabitants (n)	237	240	232

ICU, intensive care unit;

*, including also hospitals without an ICU. Data are given as absolute numbers.

## Discussion

This nationwide census identified 70 ICUs with a total of 349 ICU beds in Mongolia translating to 1.7 ICU beds per 100 hospital beds and 11.7 ICU beds per 100,000 inhabitants. Thirty-one percent of the national ICU capacity are dedicated pediatric ICU beds. In 2014, 594 ICU admissions per 100,000 inhabitants were reported. Relevant variations in the absolute number of ICU admissions per 100,000 inhabitants were observed between different provinces and Ulaanbaatar (Fig 2). Whereas the number of adult ICU admissions correlated with the number of adult ICU beds per 100,000 inhabitants, we did not observe such a relationship between pediatric ICU admission and pediatric ICU beds per 100,000 inhabitants.

The number of ICU beds per 100,000 inhabitants in Mongolia (n = 11.7) is higher than the number of ICU beds per 100,000 inhabitants in other lower-middle- and low-income countries (n = 0.1 to 4.6 [12–17]). The number of ICU beds per 100,000 Mongolian inhabitants is, for example, higher than in the United Kingdom (n = 3.5) or New Zealand (n = 4.8), comparable to some Western European countries, and lower than in Germany (n = 24.6), the United States (n = 20), Belgium (n = 21.9) or Croatia (n = 20.3) [2,18]. When comparing these numbers it is important to note that many of the aforementioned studies from high-income countries reported adult ICU beds only [18,19]. The number of ICU beds per 100 hospital beds in Mongolia (*n* = 1.7), on the other hand, appears low compared to other middle- and high-income countries (United States, n = 9; Belgium, n = 4.4; Germany, n = 4.1; Colombia, n = 3.5; Croatia, n = 3.3) [2,12–17]. One reason for this may be the relatively high number of acute care hospital beds per 100,000 inhabitants in Mongolia (n = 688) (United States, n = 221; France, n = 380; United Kingdom, n = 298; Canada, n = 300; Germany, n = 593 [18]).

Due to the lack of an international consensus on the definition of an ICU bed [19], we chose to use the definition as applied by each hospital surveyed. This definition may not be in agreement with the definition of an ICU bed as applied by other authors who consider the presence of one mechanical ventilator per bed a prerequisite for an ICU [17,18]. The finding that mechanical ventilators were available for only half of the ICU beds counted in our census suggests that the nationwide ICU capacity may have relevantly been over-estimated compared to other surveys in which a stricter definition of an ICU bed was applied.

On the other hand, the result that functional mechanical ventilators were only available for half of the ICU beds could direct at a relevant shortage of resources in Mongolian ICU services. Indeed, previous studies highlighted substantial resource restrictions in Mongolian ICUs [8,9,20]. For example, invasive blood pressure monitoring and blood gas analyzer were available in only 18.4% and 10.5% of Mongolian ICUs, respectively [19]. In comparison, a survey from Sri Lanka reported an 83% coverage of ICUs with arterial blood gas facilities [17]. No Mongolian ICU had equipment available to measure cardiac output or intracranial pressure [8,9,20]. Similarly, in 2009, no Mongolian ICU had the resources required to consistently implement the 2008 Surviving Sepsis Campaign guidelines [20].

The median number of beds per ICU was 2 for pediatric and 5 for adult intensive care services indicating that most ICUs in Mongolia are small. In provincial hospitals, this may reflect the low population density in Mongolian provinces. Interestingly, however, only few referral ICU services in tertiary hospitals have more than 10 beds. The only ICU running over 20 beds is the national referral neonatal ICU. No such ICU exists for adults in Ulaanbaatar.

A total of 17,749 admissions to Mongolian ICUs were reported in 2014. The number of adult ICU admissions per 100,000 inhabitants (n = 594) was comparable to that reported from Canada (n = 389), France (n = 426) and the Netherlands (n = 466) [18], higher than that from Malaysia (n = 108) [21], Sri Lanka (n = 194) [17] and the United Kingdom (n = 216) [18], and lower than that from Belgium (n = 1,051), the United States (n = 1,923) and Germany (n = 2,353) [18]. The number of adult ICU beds and adult ICU admissions per 100,000 inhabitants correlated (Pearson correlation coefficient, 0.5; *p* = 0.02). As our study did not evaluate the appropriateness of individual ICU admissions, we cannot conclude whether the ICU capacity in Mongolia meets the nationwide demand for adult critical care. In addition, it is important to note regional heterogeneities in ICU capacities and admission rates between different provinces and Ulaanbaatar.

About one third of the ICU capacity in Mongolia is dedicated to pediatric and neonatal cases. Two provincial hospitals even reported to have no adult but only a pediatric/neonatal ICU service. Our census identified 3.6 pediatric ICU beds per 100,000 inhabitants. This number is substantially higher than in most low- [22], middle- (e.g. Colombia, n = 0.52; Chile, n = 1.4) [23] and even high-income countries (e.g. Spain, n = 0.51; United States, n = 1.4) [24]. Only Cuba reported a higher number of pediatric/neonatal ICU beds per 100,000 inhabitants (n = 4.1) [23]. The observation that the number of pediatric/neonatal ICU beds did not correlate with the number of pediatric/neonatal ICU admissions per 100,000 inhabitants could imply that an adequate number of pediatric/neonatal ICU beds is available in Mongolia and results in variable ICU admission practices. However, it needs to be considered that these are median values for Mongolia and may not apply to all regions given substantial inter-provincial heterogeneities. Functional mechanical ventilators for children and neonates were available for less than half of the available pediatric/neonatal ICU beds. Once again this could indicate a relevant shortage of resources despite a seemingly adequate number of pediatric/neonatal ICU beds in Mongolia.

In conclusion, with 11.7 ICU beds per 100,000 inhabitants the ICU capacity in Mongolia is higher than in other low- and lower-middle-income countries. Substantial heterogeneities in the standardized ICU capacity and ICU admissions exist between Mongolian provinces. Functional mechanical ventilators are available for only half of the ICU beds. Pediatric/neonatal ICU beds make up one third of the national ICU capacity and appear to meet or even exceed the demand of pediatric/neonatal critical care.

## Supporting Information

S1 File Anonymized statistical analysis data file. (SAV)
